# Reaching the Elderly: Understanding of health and preventive experiences for a tailored approach – Results of a qualitative study

**DOI:** 10.1186/s12877-016-0374-3

**Published:** 2016-12-08

**Authors:** Christiane Patzelt, Susanne Heim, Bernhilde Deitermann, Gudrun Theile, Christian Krauth, Eva Hummers-Pradier, Ulla Walter

**Affiliations:** 1 Institute for Epidemiology, Social Medicine and Health Systems Research, Hannover Medical School (MHH), Carl-Neuberg-Str. 1, 30625 Hannover, Germany; 2 Formerly: Institute for General Practice, Hannover Medical School (MHH), Carl-Neuberg-Str. 1, 30625 Hannover, Germany; 3 Department of General Practice, University Medical Centre Göttingen, Humboldtallee 38, 37075 Göttingen, Germany; 4 Lower Saxony State Health Department, Division 4: Cancer Registry, Roesebeckstr. 4-6, 30449 Hannover, Germany; 5 Department of Radiation Oncology/Centre of Palliative Care, University of Zürich and University Hospital of Zürich, Rämistraße 100, 8091 Zürich, Switzerland

**Keywords:** Elderly, Prevention, Health promotion, Healthy aging, Target groups, Information materials, Gender, Qualitative research, Focus groups

## Abstract

**Background:**

Often preventive measures are not accessed by the people who were intended to be reached. Programs for older adults may target men and women, older adults, advanced old age groups and/or chronically ill patients with specific indications. The defined target groups rarely participate in the conception of programs or in the design of information materials, although this would increase accessibility and participation. In the German “Reaching the Elderly” study (2008–2011), an approach to motivating older adults to participate in a preventive home visit (PHV) program was modified with the participatory involvement of the target groups. The study examines how older men and women would prefer to be addressed for health and prevention programs.

**Methods:**

Four focus groups (*N* = 42 participants) and 12 personal interviews were conducted (women and men in 2 age groups: 65–75 years and ≥ 76 years). Participants from two districts of a major German city were selected from a stratified random sample (*N* = 200) based on routine data from a local health insurance fund. The study focused on the participants’ knowledge about health and disease prevention and how they preferred to be approached and addressed. Videos of the focus groups were recorded and analysed using mind mapping techniques. Interviews were digitally recorded, transcribed verbatim and subjected to qualitative content analysis.

**Results:**

A gender-specific approach profile was observed. Men were more likely to favor competitive and exercise-oriented activities, and they associated healthy aging with mobility and physical activity. Women, on the other hand, displayed a broader understanding of healthy aging, which included physical activity as only one aspect as well as a healthy diet, relaxation/wellness, memory training and independent living; they preferred holistic and socially oriented services that were not performance-oriented. The “older seniors” (76+) were ambivalent towards certain wordings referring to aging.

**Conclusions:**

Our results suggest that gender-specific needs must be considered in order to motivate older adults to participate in preventive services. Age-specific characteristics seem to be less relevant. It is more important to pay attention to factors that vary according to the individual state of health and life situation of the potential participants.

## Background

Prevention and health promotion in old age have become increasingly important in recent decades. In its report on aging and health in 2015, the World Health Organization (WHO) again emphasized the importance of healthy aging for the older population. This development would not be possible without numerous concepts to promote successful aging, launched with slogans such as active aging [1], successful aging [2–4], positive aging [5] and productive aging [6]. There is agreement regarding the multidimensionality of the concept of aging and of the view of successful aging as the result of a lifelong process [7]. The diverse criticisms include the lack of inclusion of the subjective views of the elderly and the need for diversity [8].

Social factors such as age, gender, social status, education and ethnicity affect health communication and should be considered when evaluating its ability to reach a targeted population. Sex and gender are important components of the communication process. Gender differences and other factors determine the medium and the received message of health communications [9]. Age, for example, can influence the receiver’s response to information [10]. Psychological factors such as attitudes, beliefs and values also play a critical role in the communication process. The key to successful health communication is to clearly identify the target audience based on analysis of social and psychological factors. The planning, implementation and evaluation of preventive health projects should be executed in such a way that the intended target groups can include information relevant to realizing and implementing the disseminated information in the near or distant future. The question of whether the intended form of approach and address is appropriate for the target group is another factor to consider.

Women and active people are more likely to respond to preventive health offerings and are thus easier to reach than men and older adults with multiple chronic conditions, who are considered to be rather unresponsive and hard-to-reach target groups [11]. Although there are significant gender differences in health and life expectancy as well as higher overall health risks for men, little consideration has been given to their specific needs and potentials so far [12, 13]. Prevention efforts often fail due to the lack of a target group-oriented approach. To ensure that health messages reach the elderly Löckenhoff & Carstensen [14] pointed out that it is necessary to formulate messages more relevant to older people and to tailor information to the specific needs and preferences of older people. This requires target group inclusion in the sense of participatory research. However, target groups are still rarely directly involved in the development of information materials for prevention programs.

The study “Reaching the Elderly (AeGE): Effectiveness and cost-effectiveness of different ways of reaching the elderly to participate in preventive programs drawing on the example of preventive home visits” (2008–2011) was launched in Germany with the aim of developing and optimizing target group-oriented, age- and gender-specific information materials to better motivate target groups to participate in a preventive home visit (PHV) program.

Preventive home visits (PHVs) are a type of outreach advisory service provided to older adults in their home environment. The goal of PHVs is to prevent or delay the need for long-term care, to promote health and to help older people lead an independent and self-determined life for as long as possible. The first international studies on this topic were conducted in the 1980s in Denmark, which—unlike Germany—has already adopted preventive home visits as part of the regular health care services for the elderly population [15]. Numerous other studies followed in the 1990s, especially in the USA, UK and Switzerland [16]. Löfqvist et al. [17] reviewed the existing knowledge of the substantive and formal requirements required for the development of “evidence-based” preventive home visits. There is evidence supporting the basic effectiveness of preventive home visits in old age, but there is a need to clarify the suitability of PHVs for specific target groups [18]. Preventive home visits are currently being implemented in pilot projects in Germany, where they are becoming increasingly widespread and meeting growing acceptance. However, there are great differences between projects in terms of objectives, target groups, scope and content, so a generally valid concept of preventive home visit still does not exist. The defined target groups include very old (≥80 years of age) and younger seniors (age 65 to 75 years) who do not need care as well as those who need care, live alone, have multiple chronic conditions, and are not mobile, etc. The costs are covered by a range of carriers, including health insurance companies, charities and municipalities. Therefore, the target groups and professions executing the various projects differ accordingly. In the “Healthy Aging” project organized by Local Health Care Fund (AOK) of Lower Saxony, one of the largest statutory health insurance companies in Germany, preventive health counselors (social workers, nutritionists and social scientists, etc.) have conducted preventive home visits with AOK insured persons over 65 years of age in selected regions of the state of Lower Saxony in a model project since 2004.

Older persons (65 years and older) insured by AOK were asked to evaluate the current information materials, which consisted of an information leaflet and cover letter describing the Healthy Aging preventive home visit program, for clarity, acceptance and potential to motivate participation. The written information materials used to address and approach the target groups were modified based on the results of qualitative research (focus groups and personal interviews), with additional input from an expert panel. The new and old information materials were then evaluated in two areas outside the intervention regions. In the second phase of the study, the modified information materials were introduced via two access routes (primary care physicians and the health insurance company) and evaluated for their potential to motivate participation.

In the first phase of the study, the main focus of research interest was the question of how information materials should best be designed to motivate the older adults to participate in the prevention program. It was assumed that a gender- and/or age-specific approach and language are necessary. The study analyses if these specific approaches are necessary and what characteristics should they have.

## Methods

**Fig. 1:**
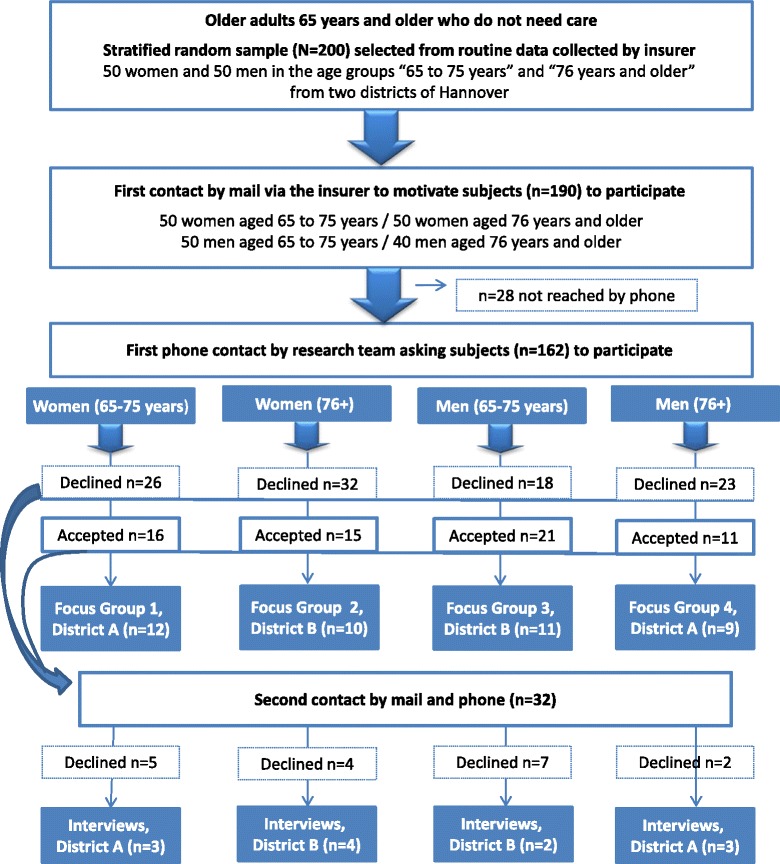
Study design

Four age- and gender-specific focus groups [19, 20] and twelve personal interviews were conducted from October 2008 to February 2009. AOK members aged 65 years and older who were living independently without the need for nursing care were sent written information materials inviting them to participate. Two districts of Hannover where preventive home visits were not yet available were intentionally selected. A sufficient knowledge of German was required for subjects to participate in the focus groups and personal interviews. The names were obtained from a stratified random sample (males and females aged 65–75 years and ≥ 76 years) from a pool of routine data collected by AOK Lower Saxony (Fig. 1).

In the review by Carlsen & Glenton [21], it was shown that the use of four to six focus groups depending on the theme and theoretical data saturation is an appropriate number. Four focus groups were conducted in the present study.

An interview guide that includes the following topics was developed for the focus groups and interviews:

1. Understanding of health in old age,
2. Experience with preventive measures,
3. Preferred way to be addressed and approached and preferred information channels.

The guide was pre-tested by a pilot focus group. Personal interviews were conducted on the basis of the focus group guide, which was expanded and slightly modified based on the results of the focus group discussions.

The study was approved by the Ethics Committee of the Hannover Medical School. In addition, the study was performed after consultation with the Data Protection Supervisor of the state of Lower Saxony in Germany. Our research was carried out in compliance with the Helsinki Declaration.

### Procedure

Older AOK members (65+) living without nursing care were selected from the stratified random sample of routine AOK data. These potential focus group participants received the written information materials from the health insurance provider. The cover letter informed them that there would be a follow-up phone call asking if they will participate or not, naming an incentive of 20 euros for participation, and a contact person at the health insurance company to answer their questions. Soon afterwards (within 1 week), participants received the follow-up call by a member of the research team and information regarding videotaping of the focus groups. Based on the experience of the research group that about one-third of insured persons who receive a written invitation participate in focus group discussions, only 40 men in the older age range were invited to a focus group in order to obtain an appropriate sample size.

After sending written information about the study, we were able to reach 162 (85.3%) out of 190 insured persons by phone. During the first contact phone call, 63 (33.2%) of the insured persons agreed to participate in one of the four focus groups (Table 1).

**Table 1:** Focus groups – Recruitment and characteristics of the participants

Focus groups	Younger target group (age 65 to 75 years)		Older target group (age 76 and older)		Total
	Women	Men	Women	Men	
Information sent	50 (100.0%)	50 (100.0%)	50 (100.0%)	40 (100.0%)	190 (100.0%)
Reached by phone, first phone contact	42 (84.0%)	39 (78.0%)	47 (94.0%)	34 (85.0%)	162 (85.3%)
Accepted	16 (32.0%)	21 (42.0%)	15 (30.0%)	11 (27.5%)	63 (33.2%)
Canceled in second phone contact	4	3	5	2	13
Excluded due to an excess of participants	0	7	0	0	7
Participated	12 (24.0%)	11 (22.0%)	10 (20.0%)	9 (22.5%)	42 (22.1%)
Duration (minutes)	100	121	111	133	Ø116
Age, participated: mean (min., max.)	70 (67-74)	68 (65-71)	79 (76-83)	83 (77-95)	
Age, non-participated: mean (min., max.)	70 (65-75)	71 (65-75)	81 (76-89)	80 (76-88)	

Reasons for refusal were documented. The reasons for refusing to participate in a focus group at first phone contact were as follows (*N* = 99):

- Health impairments (*n* = 29),
- No time (*n* = 26) due to holiday travel (*n* = 9), doctor’s appointments (*n* = 4), family caregivers (*n* = 3),
- Interested but no time (*n* = 12) due to caring for a relative (*n* = 4) and other reasons
- Not interested (*n* = 22) due to aversion to focus groups (*n* = 3), to being videotaped (*n* = 2) and other reasons
- Language barriers (*n* = 6),
- Not specified (*n* = 4).

One day before the planned focus group discussion, the participants received a phone call to remind them of the meeting and check if they still intended to participate. Reminder calls are considered advantageous due to the health issues in the target group [10]. During the second contact phone call, 13 (20.6%) of the potential participants withdrew their consent to participate. Seven men in the 65- to 75-year-old age group had to be excluded due to the large number of positive responses to the invitation in that group. The total of 42 insured persons recruited consisted of 12 women aged 65 to 75 years and 9 men aged 76 years and older from District A, and 11 men aged 65 to 75 years and 10 women aged 76 years and older from District B. The focus group discussions lasted an average of 1 h and 56 min (Table 1).

The focus group discussions were conducted in October 2008 at the community centre for senior citizens located in the respective districts of the city. All focus groups were headed by two moderators. To achieve an age- and gender-matched communication path for the target groups, the men’s groups were led by a female and a male moderator, the latter aged like the “younger seniors” of the target group, and the women’s groups were led by two female moderators.

To achieve data saturation, semi-structured interviews were conducted with an additional group of participants, who were not willing or not able to join one of the focus groups. This additional group included, in particular, immobile seniors with severe health impairments and family caregivers. Many of these individuals could not participate in the focus group discussion due to health reasons and time constraints. In January 2009, 32 individuals who had declined participation in a focus group for the aforementioned reasons, but stated that they were in principle interested in participating in the study received a second letter (Table 2). Particular effort was taken to ensure that persons living alone were included in the interviews as far as they could be identified based on the first contact information.

**Table 2:** Personal interviews – Recruitment and characteristics of the participants

Interviews	Younger target group (age 65 to 75 years)		Older target group (age 76 and older)		Total
	Women	Men	Women	Men	
Sent second letter	8	9	10	5	32
Age: mean (min., max.)	71 (65-75)	71 (66-75)	81 (76-88)	83 (79-88)	
Reached by phone	8	9	10	5	32
Accepted	3	2	6	3	14
Excluded due to an excess of participants	0	0	2	0	2
Participated	3	2	4	3	12

Of the 32 AOK members who were contacted by mail and phone a second time, 18 did not accept to be interviewed. Their reasons for refusal were as follows:

- Not interested (*n* = 5),
- No need for a personal interview; can help themselves (*n* = 4),
- Refusal by a family member (*n* = 3),
- No time (*n* = 2),
- Problematic care-giving situation (*n* = 2),
- Hospitalization (*n* = 2).

The goal was to conduct three interviews from each of the four subgroups. Therefore, a total of 12 individuals were interviewed: 3 women (65–75) and 3 men (76+) from District A, and 2 men (65–75) and 4 women (76+) from District B (Table 2). The interviews lasted an average of 45 min (range: 32 to 109 min).

### Analysis

The group discussions were videotaped with a digital recorder and subsequently analysed by our research group using knowledge mapping methodology [22]. Such mapping methods are useful, for example, for the organization of ideas and notes, for establishing a clear overview of complex issues, and for better comprehension of argumentation structures. In qualitative research, mapping methods also can be used for qualitative data structuring [22, 23]. Knowledge mapping methodology: To use this method for analysing the focus group discussions, soon after the sessions a sequential video analysis was performed in which key messages were noted on moderation cards and clustered into thematic units on meta-planning boards. Successive modifications and additions were then made. The resulting posters were photographed and the images were imported into a mind-mapping software program (FreeMind). The results were summarized, structured and comparatively displayed as mind maps. For validation purposes, the group discussions were transcribed and subjected to computer-assisted qualitative content analysis [24, 25] using the MAXQDA 2007 software program. Accordingly, the interviews were transcribed verbatim and analysed.

## Results

Below, we present the results of the focus group discussions and personal interviews on the target population’s understanding of health, subjective experiences, preferences and barriers to the use of preventive measures as well as their preferred ways to be addressed and approached and preferred information channels. In particular, the similarities and differences between the different age and gender groups are discussed.

### Understanding of health in old age

Women primarily associated health in old age with social participation, personal well-being and independent living. Men tended to have a more functionally oriented view and associated healthy aging with physical activity, mobility, and performance. “Yes, well, I have enough physical activity. Yes, I run up the stairs and down the stairs. We go for walks. That’s what we do.” (Interv 5_A_m2_00:04:45). Similarities between the two age groups were also observed. The older seniors found that the terms “Healthy Aging” and “health in old age” represented a contradiction, whereas the younger seniors did not voice this criticism of the terminology. Key aspects that the participants associated with healthy aging are listed in Table 3. The groups differed in several respects, as described below.

**Table 3:** Focus groups and interviews – Results for the question: What do you associate with “healthy aging”?

Women aged	Men aged	Men aged	Women aged
65 to 75 years	65 to 75 years	76 years and older	76 years and older
Physical activity	Physical activity	Mental and physical activity	Mental and physical activity
Healthy diet	Mobility	Social participation	Healthy diet
Relaxation	(Physical) performance	Will to live	Family embeddedness
Well-being			Independent living
Independent living			“Healthy aging” is a contradiction
Social engagement			
Social participation and communication			

### Women aged 65 to 75 years

“You’ve got to work at it” was a key message of this group of younger seniors’ understanding of healthy aging. Personal social engagement played a prominent role: “So, in other words, we should go among people. (S2) That’s right, yes! (S1) Yes. And reach out to people./Yes/Go and entertain/to form small groups, to go jogging or to make handicrafts, so, at least not remain alone, but to join a group somewhere. (S2)” (FG1_A_w1_00:11:30). These younger seniors mainly defined healthy aging in terms of social participation and communication. They listed a variety of strategies that contribute to the preservation of health and well-being: maintaining friendships, socializing, being open-minded and motivating others to become more active. They considered activities outside the family important for sustaining health. Withdrawal from social life was considered to be dangerous and to cause illness. Isolation was associated with disease.

Diet and exercise were other topics that these women associated with healthy aging. Some of female younger seniors partly stated that they implemented the knowledge that diet and exercise are important building blocks for the preservation of health in their own lives. The factors that motivated them to adopt a healthy diet were not always disease-related issues such as diabetes, but also social norms, which motivated them to lose weight and eliminate certain foods from their diet. The 65- to 75-year-old women who were interviewed emphasized the impact of genetic predisposition and previous lifestyle on healthy aging: “Yes, healthy aging is indeed, first, a personal responsibility and, secondly, also a great hit and miss. The genes that you have determine that one does not see well; you do not know what you have until it breaks at some point and you have to-well-to keep fit, and eat and drink healthy.” (Interv 1_A_w1_00:34:58). They also felt that pets contribute to a healthy lifestyle and personal well-being.

#### Men aged 65 to 75 years

These younger seniors felt that healthy aging was mainly associated with physical activity, including activities such as gardening as well as sports, for instance swimming and cycling. Regarding sports activity, this also meant testing and expanding the limits of one’s physical ability: “I associate with health not smoking and, again, I say it just once, going to ones limits. For example, I do what I can … Yes, what can I do, and sometimes I experience while biking, for example, when I bike to Hildesheim or to Celle, which is 80 km, then think oh well, then” (FG3_2_m1_00:50:46). They organized their units of sports and physical activity independently (self-management), or, in some cases, together with their wife or partner. This group also associated healthy aging with a balanced diet, not smoking and moderate alcohol consumption. Compared to their female counterparts, the male younger seniors had a concept of social participation that was not directly related to health. They emphasized the importance of independent living.

#### Women aged 76 years and older

For this subgroup of older seniors, maintaining their health was the most important issue. Above all, they associated health with independent living. They listed a number of individual strategies for preserving their physical and mental health, for example, strategies for structuring their day, memory training (e.g., with board games), puzzles, and conversations. They attempted to maintain fitness by exercising, for example, by walking and biking as well as working out on a stationary bicycle. The management of everyday life determined the daily routine of many of these women because their daily routine activities consumed more and more time: “I’m always in the garden and then I sweep again and the road is long. Now there are lots of leaves and on the street outside the foliage need to be swept up, so I have no time for something else! … I’m always and if I have time, I sit down and close my eyes sometimes. Then I’m tired and … From Monday to Saturday. (B1) … And because you are also not as fast with the hands, right? (F1)” (FG2_2_m2_00:36:58.) This group thought that healthy aging was a contradiction in terms, as illustrated by the following interview excerpts: “That’s the most important thing. To be still healthy when you’re old.” (FG2; K1: 00:18:23) … “But which old person is healthy?” (FG2; F1: 00:18:24) … “I don’t think that anybody is still really healthy when he gets old” (FG2; F1: 00:18:25).

#### Men aged 76 years and older

Older men associated healthy aging primarily with the will to live and social participation, citing interest in world affairs and community life as examples. One focus group participant responded to the question as follows: “I think the important thing is to be optimistic … to keep believing that you will stay healthy and age in peace and harmony, and that your interests will include other things besides just coping with your illnesses” (FG4, W1: 0:36:20). Mental and physical activity contributes to maintaining health and well-being. In contrast to the younger men, some of the older men thought that communicating with other people, including one’s wife or partner, was important for maintaining health.

### Preventive measures: Utilization, barriers and preferences

#### Utilization

In the younger age group (65–75 years), a number of women had considerable experience with prevention courses. The utilized services ranged from memory training and cooking classes to healthy back, pelvic floor exercise, senior citizen dancing, Qigong and autogenic training classes. Based on the wide range of services mentioned, the pattern of service utilization suggests a holistic approach.

Men in the younger age group associated preventive measures solely with exercise-related services. The older men (76+) also felt that physical exercise was important, but were less focused on group sports and more on individual exercise (Table 4), including activities together with their wives. At the same time, the men stressed that group preventive health offerings were predominantly attended by women and that men therefore had some reservations about participating. Moreover, preventive measures were associated with illness; for example, some participants stated that they had experience with healthy back classes recommended by a specialist doctor. Some felt too fit to utilize preventive services themselves-a point that suggests that prevention is understood mostly as secondary and tertiary prophylaxis and that health promotion and primary prevention services may draw less interest. The aspect of performance was also stressed in association with both independent exercise and the described experiences with preventive services such as healthy back classes.

**Table 4:** Prevention services as viewed by the surveyed target groups

Category	Women aged	Men aged	Men aged	Women aged
	65 to 75 years	65 to 75 years	76 years and older	76 years and older
Experiences	Prefer group activities	Were/are active in a sports club	No longer active in a sports club	Some; currently rather little
	Various positive and negative experiences	Focus on exercise	Health-promoting exercise on self-initiative	Active in self-initiative
		Competitive		
			Competitive	
Barriers	Fear of failure and/or injury	Classes “occupied” by women	Classes “occupied” by women	Lack of social acceptance
	Focus on performance of many groups	Association with illness	Lack of age-appropriate (sports) classes /services	Coping with daily life takes up energy
Preferences	Offerings with a holistic approach	Health insurance company contact as individual advisor	Age-appropriate social activities (e.g., organized walks)	Age-appropriate social (exercise) activities with or without spouse
	Group activities			
			Activities close to one’s home	Activities close to one’s home

In the older seniors group (76+), it was mainly the men who reported prior utilization of preventive health opportunities. The women did memory training (crossword puzzles) and played cards and/or board games at home on their own initiative, in some cases, with or for their spouse. The older men (76+) reported positive experiences when participating, for example, in a running group, a sitting exercise course (together with their wife), or memory training. Club activities and social engagement in the parish were also mentioned. They indicated that self-organized activities such as regular walks and gardening contribute to well-being. In addition, they associated preventive services with regular participation in the health and cancer screenings funded by the statutory health insurance company.

Exercise classes were mentioned in all focus groups. Swimming and water aerobics were particularly popular: many participants regularly participated in these activities, and many had already considered joining a swimming and water aerobics class or wished that these classes were available in their area.

Overall, some participants felt that their general practitioner lacked the time needed to advise patients about health promotion and disease prevention (“The doctor already has enough to do so.” (FG4_1_m2_01:23:27) and/or thought that this advisory role was not the job of a doctor (“Health insurance. The insurance company would have to offer us that.” (FG4_1_m2_01:22:46). In their opinion, doctors are responsible for disease treatment and not for health promotion. Only one of the younger men aged 65 to 75 years felt that primary care physicians should be seen as “prevention guide” because they have the competence and knowledge of patients’ history required to inform their patients what they could do.

#### Barriers

The barriers to the utilization of health promotion and disease prevention offerings were very diverse. The men listed formal criteria, such as insurance concerns (older men aged 76 years and older), financial aspects and the time and effort required to go to the health insurance office (younger men aged 65 to 75 years), but also the preponderance of women in preventive health activities: “Since there are usually 99.9% women, men find it very difficult [to participate].” (FG3; Sch: 01:06:02). They also mentioned the decrease in activity with age and the steadily decreasing size of their peer group.

The women were somewhat more specific: Women in the younger age group (65 to 75 years) indicated that because of fear of (physical) failure and their rejection of competitive activities, the offered services often did not meet their needs. Women in the older age group (76+) stressed that the efforts of everyday life, the feeling of decreasing strength and intensive family responsibilities were already enough of a challenge. The fear of being injured in sports and physical activities was another issue. Furthermore, some of the activities that they might have enrolled in were not socially accepted. One woman said, “I once told my children that I wanted to start working out and they nearly fell over laughing.” (FG2; S1: 00:27:14).

#### Preferences

The participants’ specific wishes regarding the design of health promotion and disease prevention opportunities were discussed in the focus groups. Men and women in the younger seniors groups indicated that there should be offerings tailored to one’s specific health condition. Group course offerings for men should be different from those for women. When addressing men the functionality of an offering should be emphasized.

The older men (76+) expressed a desire for age-appropriate services from which they could pick and choose. This age group placed emphasis on potential social offerings, e.g., hiking groups. The older women (76+) wanted active exercise classes with opportunities for socializing (e.g., fitness, swimming and dancing), but said that the classes should focus more on health preservation and that socializing should be a fringe benefit. The women indicated that it was important to have opportunities close by, and that it should be possible for them to utilize these services together with their partner.

### Approach and address preferences and preferred information channels

Focus group participants and interviewees were asked how and in what way they wished to be approached and addressed. They disliked general mailings. They preferred targeted mailings in which they were personally addressed (Mr., Mrs. or Ms. X). Furthermore, they wanted the personalized mailing to be sent in a sealed envelope and to also be addressed to their spouse, if appropriate.

They indicated that it would be okay to inform them about the program by phone soon after they received prior written notice. However, they stressed that people should be given sufficient time to thoroughly read and consider the information. Sole phone contact was generally disliked, especially by the older seniors. The individuals surveyed expressed several concerns, for example, that the pressure of having to make quick decisions on the phone could be overwhelming. “So I would prefer to be informed specifically by written notice. … I ended up lying there. I can read, I’ve got all day. Then they can leave me two days time to answer.” (FG2; P2: 00:42:01).

In their opinion, the health insurance company would be a more appropriate provider of information on preventive home visits than a general practitioner or specialist physician. They wanted the health insurance company to offer individualized and disease-specific preventive health opportunities.

In the younger seniors group, women and men in particular emphasized the importance of self-initiative and described the ways by which they searched for information. Some went to the health insurance office directly to request information and take home brochures. In the older seniors group, men were more likely to only read mailings and newsletters they received from the health insurance company (passive behavior). For older women, the main source of information was not the health insurance company but community centres (parish, social meeting place) and local senior citizens services. The older women also took advantage of “open house” events sponsored by pharmacies to obtain information. As a group, women listed newspapers and friends as their main sources of important information about health promotion and disease prevention opportunities. Men, on the other hand, mainly received such information from their doctors and health insurance companies (active and passive information gathering). But also the spouses were an important source of health-related information.

Television and radio were not considered to be sources of information about preventive health opportunities. There was hardly any awareness of the Internet as a potential source. It was only used by a small number of younger seniors and only one of the older seniors. Both groups relied on their children and grandchildren for information from the Internet.

This research made it clear that only a few older adults are aware of the preventive health opportunities available to them. Health insurance company members’ magazines, local newspapers and drugstore newspapers seem to be the best channels for targeted information dissemination. Appropriate color highlighting of the information is particularly important for this target group. It is also important to make information about preventive health opportunities offered by health insurance companies better known (e.g., via mailings) and easier to access.

## Discussion

The present study showed gender differences regarding the understanding of health and ways one wished to be informed and approached which suggest the need for a gender-sensitive approach. Men associated health and preventive health opportunities with physical activity and mobility in the context of competitive activities. Emphasizing similar aspects (physical fitness and effort) in an intervention study (seniors’ exercise courses) achieves higher rates of male participation [26, 27]. This high male participation rate of approximately 48.0% can be attributed to the target group-specific method of address and approach, gender-specific course program planning, and the combination of multiple target group-specific access channels, such as general practitioners, institutions, health insurance companies, newspapers, acquaintances and friends.

The deciding factor of motivation for men’s participation in sports and physical activity is performance [28]. Men frequently expressed declining performance with age as a concern. Women tended to list weight gain, beauty standards and/or pressure to be slim as the deciding factors. This was also reflected in our study.

The interview study by Hartmann-Tews et al. [28] identified motivational differences in the different age groups: Older seniors were more likely to name personal responsibility and moral obligation to maintain one’s health as their motivation for participating in preventive health sports and physical activities, whereas the enjoyment of sports and physical activity was the main motivation for younger seniors. Our study participants were not explicitly surveyed on these reasons and motivations.

As a whole, women in our study displayed a broader understanding of healthy aging than men. Women considered physical activity as only one aspect in addition to a healthy diet, relaxation/wellness, memory training and independent living. As well preventive behavioral courses for relaxation, diet and exercise tend to find greater acceptance among women. These topic areas comply with their self-image in gender roles. Thus, persons who utilize preventive health offerings should find this reflected in mailings and the structure of the offerings. This is most likely when they are confronted with gender stereotypes that reflect their self-image [29–31].

Our results are conformity with those of Jopp et al. [32], who examined the lay perspective of young, middle-aged, and older adults from the United States and Germany concerning successful aging. According to their study, a broad understanding is only associated with female sex and (not carried out in our study) higher education, but not with age. Overall, laypeople viewed successful aging in far more multidimensional terms than those in scientific theories [26, 29, 33]. Remarkable differences between the two countries were also observed. These discrepancies can be explained by the slow start of dissemination of the concept among the German public. Another striking feature is the problem particularly older people (Germans) have with the terms “healthy aging”, “health in old age” and “health promotion”; in their view, these terms are apparently incompatible with the health restrictions that they already have. Other studies also reflect these findings [28]. In practice, numerous preventive health services with these titles are (still) being offered, at least in Germany. Based on the results our this study, the project title of the Preventive Home Visit program was changed from “Healthy Aging” to “Independent Living in Old Age” [33], which was proposed by older persons, themselves.

Our study highlights the need for preventive interventions tailored to the needs of elderly individuals and designed according to the rules of participatory research from the outset. The present study showed that the inclusion of older people provides a wealth of information useful for designing materials to address specific target groups. Thus, they help to ensure that all information requirements (increased relevance, inclusion of affected parties, consideration of possible stressors, etc.) will be met [34].

Our results are supported by quantitative and qualitative studies, which underline differences in multimorbidity patterns [35] and preventive behavior by age and sex/gender. A systematic review by Dryden et al. [36] explored the socio-demographic, clinical and social cognitive characteristics of those who do and do not engage with general health checks or preventive health checks for cardiovascular disease. The included 39 quantitative and qualitative studies consistently indicate that males are less likely to engage with health checks or screening and to endorse periodic health examinations than females. In general, attenders at health checks are older than non-attenders (10 studies), although 6 studies found no association between age and attendance. Nevertheless, the heterogeneous nature of the study methodologies meant that it was difficult to define an optimum age for uptake. Indeed, the relationship between age and participation may not be linear. The systematic review by Sun et al [37] included 53 published reports of original research that independently reported: the physical activity level of non-institutional older adults (aged 60 years and over); and the proportion of older adults in the different samples who met physical activity recommendations or guidelines. Older age groups were less likely than the reference group to be regularly active, and women were less likely than men to achieve regular physical activity, especially leisure time physical activity, when measured by both subjective and objective criteria.

There are increasing demands for the consideration of sex and gender in studies [38] and for the gender-sensitive design of prevention programs. In 2015, German insurance companies were required by law to make account for gender-specific differences when providing health care and prevention services (Code of Social Law V, Prevention Act). The present study provides one of first steps for this.

Regarding the limitations of our study, it is highly probable that most of the subjects who participated in the focus groups and personal interviews were persons interested in the topic of health in old age, and that unmotivated and disinterested individuals were less likely to participate (selection bias). Therefore, the large number of preventive health opportunities utilized and the small number of barriers to participation in preventive health opportunities must be analysed with caution. It is possible that our data over- or underestimate the actual figures. A strength of this study was the additional personal interviews conducted in the home environment which allowed us to recruit hard-to-reach persons who otherwise would have been unable to participate due to immobility, the burden of caring for family members, or reservations about participating in focus groups. Focus groups and personal interviews were used as complementary methods because the focus group method did not reach all of the target groups. Regarding the different methodological approaches, the opinions of an individual may be overheard or never raised in the group discussions in focus groups. Personal interviews, on the other hand, are limited in the sense that interviewees are limited to their own opinions and ideas, whereas the ideas of an individual can be further developed and refined in focus group discussions.

Regarding the initial research questions, the results of our focus group and interview study indicate that to enhance participation, written information materials for men must address different aspects than those for women. Differentiation between younger and older seniors does not seem to be necessary. Instead, our results suggest that it is important to regard *individual* factors that vary according to the state of health and life situation of the individual. Age alone is not the sole deciding factor. Based on these findings, in the second phase of the study a questionnaire for target group identification was developed: IboPräv - Identifikationsbogen Präventiver Hausbesuch (Identification Form for Preventive Home Visits) [33].

By designing and evaluating age-specific and gender-sensitive written approach materials based on the results of the first phase of the study, it was possible to achieve the target group-specific form of address and approach that is often required but neglected in research and practice. In the further course of the study and project, the target group-specific information materials (letter, flyer) developed in a multistage process were used to motivate AOK members over 65 years of age to participate in the preventive home visits program.

## Conclusions

Our study underlines the relevance of gender-specific preventive approaches. According to the recommendations of the participatory research the integration of the elderly from starting the concept planning boosts the target accuracy. Further research is needed to show to what extent a need-oriented differentiation is necessary.

## Abbreviations

AeGE: Acronym for the study “**Ae**ltere **G**ezielt **E**rreichen” (Reaching the Elderly)
AOK: Local health care fund
PHV: Preventive home visit program
